# An Extemporary Letter Concerning Some Physiological Curiosities

**Published:** 1783

**Authors:** 


VIII. *Lettera estemporanea sopra alcune curiosita fisiologische.* i. e. *An extemporary letter concerning some physiological curiosities.* 8vo. 1782. 18 pages.

THIS letter, which is signed " Il Cavalier Michela Rosa," is addressed to Monsignor Garampi the Apostolic Nuncio at Vienna, to whom the author, who is profeſſor of the practice of phyſic in the univerſity of Modena, acknowledges his obligations for enabling him to perform experiments on large animals.

Since the publication of this firſt letter the author has addreſſed a ſecond on the ſame ſubject, to the ſame prelate, and a third to Comte Greppi. Of theſe two we have ſeen only a French tranſlation in manuſcript, which, together with the printed copy of the firſt letter, was obligingly put into our hands by Sir Joſeph Banks, Bart. whoſe zeal for the improvement of ſcience in general leads him to procure early information of every thing that is going on amongſt philoſophers in different parts of Europe, and who was deſirous that the facts contained in theſe letters might be candidly examined.

In the firſt letter the author obſerves, that in the dead animal almoſt all the blood is found in the veins: hence he concludes, that during life there is but little in the arteries, the veins being ſufficient to contain all the blood.

He ſuppoſes the arteries to be filled with what he calls an expanſible animal vapour (*vapore eſpanſile animale*) very different from the *halitus ſanguinis* of Haller, and which he thinks is acquired quired in the paſſage of the blood through the lungs.

He conſiders this vapour as the animating principle of phyſical life, as the agent and chief inſtrument of all our functions. Filtered through the extremities of arteries, he ſuppoſes that it paſſes into the different ſecreting organs, and communicates energy to them. He conſiders it likewiſe as the principle of nutrition.

He aſſerts, in oppoſition to the experiments of Dr. Darwin and others, that the arteries and even the veins are diſtended when placed under the exhauſted receiver of an air pump.

He trembles, he ſays, when he conſiders that he is to be the firſt to announce, that the only veſſels deſtined to form the ſanguineous ſyſtem are the veins, and that the blood is not intended by nature to remain in the arteries. "As it is "by violence as it were (ſuch are his expreſ-"ſions) and contrary to its natural tendency that "the blood is driven by the heart into the arte-"ries, ſo it haſtens to eſcape from thence, as "from a reſidence which is foreign to it." If nature has given more ſtrength to the right ventricle of the heart than to the left, and a greater diameter to the arteries that paſs through the lungs than to the veins, it is, he thinks, in order that that the blood may be more expanded, and of courſe more eaſily and ſooner impregnated with the vital flame, as he calls the vapour in queſtion.

In the SECOND letter after proving from different writers that the arteries after death are found almoſt entirely empty, he aſſerts, that if we divide the carotid artery of a living ſheep, calf, or bullock, the blood will eſcape with an inſtantaneous hiſſing noiſe, like an exploſion.

Having contrived to fix a canula in the carotid of a calf, and to make it communicate with the exhauſted receiver of an air pump, the blood, he tells us, frothed much; while the veſſel became filled with a cloudy vapour.

In another experiment a bladder was fixed to a tube communicating with the carotid of a living bullock, and when filled with blood, was tied without its contents having had any communication with the external air. This bladder, as the author aſſerts, expanded upon being immerſed in warm water.

The ſwelling of the cutaneous veins after violent exerciſe he aſcribes to their becoming arterial, or, in other words, to their being filled with vapour.

He contends that the pulſation of an artery does not depend on its coats, but on the vital air air it contains; and alſo that an artery neither dilates nor lengthens itſelf when it beats.

In his THIRD letter the author aſſerts, that in the living animal he has conſtantly found the iliac, pulmonary, and carotid arteries empty. He obſerves, that if the arteries were completely filled with blood there could be no circulation. He aſks how the effects of fear or cold in occaſioning paleneſs can be explained on the commonly received principles that the blood in ſuch caſes is driven from the cutaneous veſſels towards the heart, if the arteries are all equally diſtended? Laſtly, he tells us he transfuſed five pounds of blood into a ſheep, and yet the animal lived two hours after the operation. This he deems a proof that the arteries were not full before.

Such are the principal arguments contained in the Chevalier Roſa's three letters. The hypotheſis they are intended to ſupport, viz. that the arteries are filled with air inſtead of blood, is as old as the days of Praxagoras, and was long ago ably and completely refuted by Galen. In theſe enlightened times we little expected to ſee the dreams of the ancients on this ſubject revived, and vindicated by concluſions drawn from miſtaken notions of phyſiology, and by experiments which will not ſtand the teſt of repetition. As a proof of what we have advanced, it will be ſufficient to obſerve for the information of our readers, that Dr. Simmons, and Mr. Cline, after reading profeſſor Roſa's letters, made the following experiments in Mr. Cline's anatomical theatre at St. Thomas's hoſpital in London.—1. After laying bare the carotid of a living rabbit, they paſſed two ligatures round it, ſo as to include a portion of artery of about half an inch in length. This portion of artery was then taken out and placed under the receiver of an air pump, the air of which was drawn out without producing the leaſt appearance of diſtention in the blood veſſel. The piece of artery was then opened and found to be diſtended with blood.—2. In a like trial with a portion of the vena cava, the reſult was exactly the ſame.—3. A ſimilar portion of the iliac artery of the ſame animal was then diſtended with air, and this when placed under the exhauſted receiver expanded, as was expected, to at leaſt three times its natural bulk.——Theſe gentlemen were aware that accounts of ſimilar experiments are upon record,[†] but in examining the validity of the facts alledged by the Chevalier Roſa, they

+ Made by Dr. Darwin. See Phil. Trans. V. 64. and Ed. Med. Com. V. IV. p. 34.

chose rather to appeal to their own experience than to books.

